# Early Postural Instability with History of COVID-19 Influence Related to Diabetes: An Exploratory Cross-Sectional Study

**DOI:** 10.3390/jcm15093178

**Published:** 2026-04-22

**Authors:** Kathrine Jáuregui-Renaud, José Adán Miguel-Puga, Aida García-López, María de Lourdes Tirado-Mondragón

**Affiliations:** 1 Unidad de Investigación Médica en Otoneurología, Instituto Mexicano del Seguro Social, Ciudad de México 06720, Mexico; adan.miguel@imss.gob.mx (J.A.M.-P.); draidagarcia@gmail.com (A.G.-L.); 2 Hospital General de Zona 8, Instituto Mexicano del Seguro Social, Ciudad de México 01090, Mexico; marilutm16@hotmail.com

**Keywords:** diabetes, postural stability, COVID-19, muscle strength, ankle

## Abstract

**Background/Objective:** In late adulthood, the increasing prevalence of diabetes overlaps with the highest prevalence of postural instability. A cross-sectional study was designed to explore the combined influence of age, gender, history of COVID-19 quadriceps strength, and Body Mass Index (B.M.I.) on the postural stability of adults with/without diabetes, under a variety of sensory conditions. **Methods:** A total of 263 adults aged 21 to 82 years old accepted to participate, 99 with and 164 without diabetes. They had no history of vestibular/otology/neurology/autoimmune/orthopedic disease or proliferative retinopathy/severe renal dysfunction/traumatic injury. After clinical and vestibular evaluations, postural sway was recorded on hard/soft surface, eyes open/closed, and without/with 30° neck extension. Bivariate analysis and repeated measures multivariate analysis of covariance were performed with 0.05 significance. **Results:** In the two groups, two thirds of the participants had excess weight and almost half had history of COVID-19. Overall conditions, gender and diabetes were the main factors contributing to sway area (multiple R = 0.28–0.31, *p* ≤ 0.001) and to sway length (multiple R = 0.34–0.47, *p* ≤ 0.00001). Compared to adults without diabetes, in those with diabetes, the age was not related to sway measurements; with contribution to sway from history of COVID-19 and quadriceps strength, and decreased contribution of the study variables to both the anterior–posterior position of the center of pressure and ankle movement (velocity as a function of the anterior–posterior position of the center of pressure) (*p* > 0.05). **Conclusions:** Diabetes may interfere with the influence of individual cofactors contributing to postural sway, including decreased influence of age and reduced ankle movement. A history of mild–moderate COVID-19 may have influence on postural control in varied sensory conditions.

## 1. Introduction

Postural stability in upright stance is essential for adequate performance of activities of daily living. Accordingly, a multifactorial negative association exists between physical activity and dizziness [1], which is mainly related to vestibular and cardiovascular diseases [2]. Though the specific diagnoses vary across the lifespan, the general prevalence of these disorders increases with age [2,3].

During upright stance, to prevent falling, the body’s center of gravity is upheld within the limits of stability by the ankle torque in the anterior–posterior (A.P.) plane, and by the hips in the lateral–lateral (L.L.) plane. To estimate the body’s center of gravity, a standard method of assessing postural stability is to record the displacement of the center of pressure of the feet (posturography), during a variety of sensory conditions, such as closing the eyes (with/without vision), or to stand on a soft surface (distorted somato-sensation) [4], or neck extension (position of the utricles) [5]. The main posturography measurements are sway area, sway length, A.P. and L.L. position of the center of pressure; nonetheless, the velocity displacement of the center of pressure in the A.P. plane (V.F.Y.) is useful to monitor the ankle movements of short length–high velocity [6].

The preservation of postural stability involves complex processes, including sensory integration and motor control [4], which allow adaptation to both the inertial forces acting on the body and the inertial features of the body (e.g., weight, height). The intricacy of these processes implies that several cofactors contribute to interindividual variability. Postural stability may decrease during adulthood, with greater deterioration from the 7th decade of life; although females may display better stability compared to men (*n* = 7979, aged ≥30 years) [7]. Consistently, from the 4th decade of life, muscle strength of the dominant leg may decrease (346 men/308 women, aged 20 to 93 years old) [8]. High Body Mass Index (B.M.I.) may also contribute to postural instability irrespective of gender [9,10].

Among other health conditions, COVID-19 has also been related to postural instability, regardless of disease severity and age [11,12], with impaired postural control and physical capacity [13], as well as decreased muscle mass and strength in adults with long COVID [14]. In addition, evidence has shown a bidirectional relationship between COVID-19 and diabetes mellitus (diabetes). In a systematic review of 729 studies (*n* = 29,874,938), the pooled prevalence of diabetes in stratified COVID-19 groups was 14.7% (95% Confidence Interval, C.I. 12.5–16.9%) among confirmed cases, and 10.4% (95% C.I. 7.6–13.6%) among non-hospitalized cases [15].

Although the global rate of complications of diabetes is unclear [16], the incidence of diabetes is increasing around the world, particularly in low- and middle-income countries [17]. In 2024, the estimated global prevalence of diabetes in adults aged 20 to 79 years old (246 studies) was 11.11% (95% C.I. 11.05–11.17%), while by year 2050 it is projected to be 12.96% (12.91–13.02%) [16], with increasing occurrence of early-onset diabetes (age <40 years) accompanied by high rate of complications [18].

In adults with diabetes, postural instability has been mainly related to multisensory deficits and musculoskeletal deterioration, including accelerated loss of muscle strength, volume, and quality over time [19]. Particularly, diabetes is related to marked reduction in the muscle strength of the lower leg [20,21]. Although evidence has shown increased postural instability in adults aged 70 to 80 years old [22], studies including a wide age interval are scarce. Nonetheless, the prevalence of high B.M.I. is particularly significant in patients with diabetes [23]; a systematic review showed that obesity rates exceeded 30% in 38 of 44 studies and 50% in 14 of 44 studies [24]. In addition, over recent decades, increased higher B.M.I. has been related to increased youth onset of diabetes [25,26]. However, the combined effects of these factors on postural stability are uncertain.

The increasing prevalence of diabetes [17] that is related to high B.M.I. [23] predisposes to SARS-CoV-2 infection [27] and musculoskeletal deterioration [19], which requires attention. Each of these factors can contribute to postural instability [8,9,10,13,28], along with the high prevalence of postural instability in adulthood that may vary according to gender [7]. However, studies assessing interactions among all these factors are scarce.

The interplay among cofactors contributing to postural control is relevant for both adequate evaluation and clinical judgment. The aim of this study was to explore the combined influence of age, gender, history of COVID-19, quadriceps strength, and B.M.I. on the posturography recordings of adults with/without type 2 diabetes, under a variety of sensory conditions.

## 2. Materials and Methods

### 2.1. Participants

After authorization by the Institutional Research and Ethics Committees (IMSS R2018-785-046, 29 May 2018), a total of 263 adults (21 to 82 years old, 156 women/107 men) provided their written informed consent to participate in this cross-sectional exploratory study. They were 99 with type 2 diabetes (no other type of diabetes) that was confirmed by their family physician at the Mexican Social Security Institute, and 164 without diabetes according to their clinical records (patient’s relatives/companions) (Table 1). The two groups were invited to participate consecutively and simultaneously, according to the same selection criteria, at the Mexican Social Security Institute, from February 2023 to December 2025.

Participants in the two groups were selected because they had no history or clinical records of vestibular/otology/neurology/autoimmune/orthopedic or severe renal disease, or proliferative retinopathy, postural hypotension, exposure to ototoxic medication, injurious noise levels, or traumatic injury. At the very end of sampling, <5% of the participants without diabetes were selected to ensure the age matches for the oldest participants with diabetes.

The sample size of 99 participants with diabetes was calculated considering [29]: 8 repeated measures of sway length, with standard deviation of 100 mm and difference of 30 mm [28]; 0.5 expected correlation among repetitions; two-tailed 0.05 type I error and 0.20 type 2 error. Two more participants with diabetes accepted to participate in the study, but they did not complete the study protocol due to claustrophobia during vestibular evaluation.

### 2.2. Procedures

After clinical assessments (including peripheral neuropathy and retinoscopy) and vestibular evaluation, measurements were performed to estimate B.M.I., quadriceps strength, and postural sway on hard/soft surface, either with eyes open/closed, or without/with 30° neck extension.

To assess peripheral neuropathy, the Michigan Diabetic Neuropathy Score [30] and Semmes–Weinstein 10 g monofilament test [31] were administered to all participants; subsequently, when any of these instruments was positive, nerve conduction was appraised (Spirit, Nicolet, Madison, WI, USA).

To estimate the B.M.I. (kg/m^2^), body weight and height were measured.

Quadriceps isometric strength was estimated by the average of 5 attempts (right/left) in a sitting position (Baseline, Back-leg-chest dynamometer, White Plains, NY, USA); for the statistical analysis, the average of the dominant leg was used.

Vestibular evaluation included clinical examination and eye movement recordings, with angular vestibulo-ocular responses (V.O.R.) to sinusoidal rotation at 0.16 Hz and 1.28 Hz (60°/s peak) (I-Portal-NOCT-Professional, Neuro-Kinetics, Pittsburgh, PA, USA).

Static Posturography was performed at a sampling rate of 40 Hz (900 points/kg resolution, 16b analog to digital converter) (Posturolab 40/16, Medicapteurs, Cedex, Balma, France). Subjects were asked to stand upright as still as possible with arms at their sides, while barefoot, with 30° lateral rotation between the feet and 3.5 cm heels apart. Recordings were made for 51.2 s, either with eyes open or closed, with/without 30° neck extension, and while adding or not on a layer of foam rubber (5 cm thick, density of 2.5 p.c.f.) to the base of support. The sensory conditions were 1. hard surface with eyes open; 2. hard surface with eyes closed; 3. hard surface with eyes open; 4. hard surface with eyes open and 30° neck extension; 5. soft surface with eyes open; 6. soft surface with eyes closed; 7. soft surface with eyes open; 8. soft surface with eyes open and 30° neck extension. The order was the same for all participants, starting from the condition that is similar to activities of daily living (i.e., eyes open/hard surface/without neck extension) to the most unusual condition (i.e., eyes open/soft surface/neck extension).

### 2.3. Statistical Analysis

Data distribution was assessed by Kolmogorov–Smirnov test. Accordingly, an exploratory bivariate analysis was performed using either nonparametric statistics (Mann–Whitney U test and Spearman’s correlation coefficient) or parametric statistics (Student *t* test and Pearson’s correlation coefficient). Comparisons between proportions of the two groups were performed using *x*^2^ test. Then, considering background knowledge and the results of the bivariate analysis on the eight sensory conditions, repeated measures multivariate analyses of covariance were performed on each posturography measurement using augmented backward elimination, with the multiple R estimation for each posturography measurement (across sensory conditions), with a significance level of 0.05.

## 3. Results

### 3.1. General Characteristics of the Participants

The age and the female/male ratio were similar in the two groups (*p* > 0.05). Obesity (B.M.I. ≥ 30) was prevalent in participants with diabetes (51.1% with versus 31.1% without diabetes; *x*^2^ = 10.37, *p* = 0.001), while overweight (B.M.I. 25 ≤ 30) prevailed among participants without diabetes, with no significant difference between the groups (*p* > 0.05). All of the participants, except for one without diabetes, reported excess weight gain during adulthood.

In the two groups, tobacco use was not frequent (≤15%) and alcohol intake was reported mainly by men (≤30%), with no heavy drinkers; less than half of the participants reported the use of spectacles due to refraction errors. Compared to participants without diabetes, participants with diabetes had lower quadriceps strength (Table 1).

### 3.2. Clinical Background

About half of the participants either with or without diabetes had a history of COVID-19 (Table 1). However, none of them had severe or critical COVID-19, and none of them were diagnosed with long-COVID. In the two groups, the frequency of hospitalization was low, two (2.0%) participants with diabetes and seven (4.2%) participants without diabetes. In the two groups, vaccination was similar, with a median three shots (Quartile 1–Quartile 3 = 3–4) in participants with diabetes and median three shots (Q1–Q3 = 3–3) in those without diabetes; however, vaccination was provided according to a national plan with no institutional record of the type of vaccine that was administered to each participant.

One third of the participants with diabetes and a fifth of participants without diabetes had systemic high blood pressure, while dyslipidemia was less frequent in the two groups; however, compared to participants without diabetes, participants with diabetes had higher frequency of both systemic high blood pressure and dyslipidemia (Table 1). None of the participants without diabetes was positive for peripheral neuropathy, while 54% of men (95% C.I. 36–71%) and 33% of women (95% C.I. 21–45%) with diabetes had peripheral polyneuropathy.

Among the participants with dyslipidemia, 9 (9.0%) participants with diabetes and 10 (6.0%) without diabetes were medicated with statins, with no significant difference between the groups (*p* > 0.05). In participants with diabetes, 74 (74.7%) were medicated with metformin and 25 (25.2%) with other antihyperglycemic agents alone or combined with insulin, which was administered to 20 (20.2%) participants.

### 3.3. Posturography

#### 3.3.1. Exploratory Bivariate Analysis

Measurements by gender during each sensory condition are described in Table 2. In most sensory conditions, the sway area and the sway length were larger in participants with diabetes compared to those without diabetes except for the sway length in conditions 2 and 6, which were recorded with eyes closed, either on hard or soft surface. Additionally, a V.F.Y. reduction trend was observed in participants with diabetes during conditions 5 and 7, which were recorded with eyes open on soft surface.

Within the group of participants with diabetes, the expected correlation between the age and the A.P. position of the center of pressure or the V.F.Y. was not evident, while in participants without diabetes, these relationships were consistent, for both the A.P. position of the center of pressure (rho = 0.21–0.37, *p* ≤ 0.01) and the V.F.Y. (rho = 0.22–0.41, *p* ≤ 0.005). A similar result was observed for these two sway measurements and the B.M.I., with no evident correlation in participants with diabetes, but moderate correlation in participants without diabetes, for the A.P. position of the center of pressure (rho = 0.23–0.31, *p* ≤ 0.003) and the V.F.Y. (rho = 0.18–0.30, *p* ≤ 0.02).

In contrast, the correlation between quadriceps strength and the A.P. position of the center of pressure was evident in participants both with diabetes (rho = 0.20–0.35, *p* ≤ 0.05) (except for conditions 3 and 4) and without diabetes (rho = 0.40–0.45, *p* < 0.00001); also, the correlation between quadricep strength and the V.F.Y. was evident in participants both with diabetes (rho = 0.20–0.34, *p* ≤ 0.04) (except for conditions 2, 4 and 6) and without diabetes (rho = 0.15–0.42, *p* ≤ 0.05).

Additionally, in participants with diabetes, a relationship between the V.O.R at 0.16 Hz and the sway area was observed just for condition 1 (rho = 0.21, *p* ≤ 0.03), while in participants without diabetes, it was evident during conditions 1 and 2 (rho = 0.18–0.23, *p* ≤ 0.02).

In all participants (*n* = 263), simple correlation was low or low to moderate between age and: sway length (rho = 0.14–0.26, *p* ≤ 0.02), A.P. position of the center of pressure (rho = 0.15–0.21, *p* ≤ 0.01) and V.F.Y. (rho = 0.12–0.30, *p* ≤ 0.03), as well as between the B.M.I. and A.P. position of the center of pressure (rho = 0.20–0.23, *p* ≤ 0.001) and V.F.Y. (rho = 0.14–0.20, *p* ≤ 0.02) (except for condition 5), between the quadriceps strength and A.P. position of the center of pressure (rho = 0.31–0.37, *p* ≤ 0.00001) and V.F.Y. (rho = 0.13–0.38, *p* ≤ 0.02) (except for condition 2), and between the V.O.R. at 0.16 Hz and sway area (rho = 0. 12–0.21, *p* ≤ 0.03) (except for conditions 4 and 6).

#### 3.3.2. Multivariate Analysis

The repeated measures multivariate analysis of covariance within each group showed that a contribution to variance from gender was evident in participants with and without diabetes; on the contrary, a contribution from age was evident just in participants without diabetes and not in participants with diabetes (Table 3 and Table 4). In participants with diabetes, the B.M.I. was related to the A.P. position of the center of pressure, while in participants without diabetes, it was related to both the A.P. position of the center of pressure and V.F.Y. In participants with diabetes, the quadriceps strength was related to both the sway area and the sway length, as well as to the V.F.Y., while a history of COVID-19 was related to the sway area, the sway length and the L.L. position of the center of pressure (Table 3). In contrast, in participants without diabetes, a history of COVID-19 was related just to the differences among sensory conditions, but not to the measurements recorded during each condition (Table 4). No other consistent relationships were observed, including those related to the clinical characteristics of the participants with diabetes.

The summary results of the repeated measures analysis in all participants are described in Table 5. Gender was the only factor consistently associated with sway measurements across conditions, with beta values of 0.19–0.34 (*p* ≤ 0.03) for sway area; 0.22–0.40 (*p* ≤ 0.01) for sway length; 0.20–0.25 (*p* ≤ 0.03) for L.L. position of the center of pressure (just for conditions 1, 2 and 6); 0.38–0.50 (*p* ≤ 0.000001) for A.P. position of the center of pressure; and 0.17–0.31 (*p* ≤ 0.03) for V.F.Y. (just for conditions 1, 3. 5 and 7). Also, across conditions, diabetes was mainly related to both sway area and sway length, with beta values of 0.19–0.26 (*p* ≤ 0.002) for sway area, and 0.15–0.25 (*p* ≤ 0.02) for sway length (except for condition 2).

The posturography measurements showing differences among conditions were sway area, sway length and V.F.Y., with consistent influence from gender, and influence of diabetes on both sway area and sway length. No interaction between gender and diabetes was observed. Of note is that the age was related to sway length and V.F.Y., with beta values of 0.19–0.25 (*p* ≤ 0.003) for sway length, and 0.14–0.24 (≤0.02) for V.F.Y. (just for conditions 1, 2, 3, 5 and 6). No other consistent relationships were observed.

## 4. Discussion

This study aimed to assess the influence of age, gender, a history of COVID-19, quadriceps strength, and B.M.I. on the posturography recordings of adults with/without diabetes. Across sensory conditions, analysis within each group showed group differences on the correlation between the study variables and postural sway. The relationship between age and sway was not evident in participants with diabetes, nor was the relationship between B.M.I. and the V.F.Y. On the other hand, mainly in the group of participants with diabetes, quadriceps strength and a history of COVID-19 (regardless of gender) were related to postural sway. In the full sample, gender and diabetes were the main factors contributing to the sway area and the sway length.

In agreement with previous studies [28,32,33], in this study, participants with diabetes exhibited larger postural sway than participants without diabetes. However, regardless of diabetes, gender was also related to postural sway. In the two study groups, the area and the length of sway were larger in males than in females. This result is consistent with the evidence that, among children of the same age, girls may show better postural stability than boys [34], while adult males may display more sway during static upright stance than females [7], particularly at old age [7,35].

The contribution of age to the variance on the length of sway and ankle movement in participants without diabetes is consistent with the evidence that postural stability decreases with aging [7]. Although a previous study showed increased postural instability in adults mainly 70 to 80 years old [22], in this study, the wide range of age of the participants permitted us to observe that younger adults with diabetes may display postural instability that is unrelated to age but is instead related to several cofactors, which suggests anticipated aging.

In participants with diabetes, among the cofactors contributing to postural instability, decreased quadriceps strength was evident in both men and women (Table 1). In a previous study, quadriceps strength mainly had influence on the postural response to challenging conditions [28]. In this study, the reduced ankle movement while standing on soft surface in participants with diabetes suggests that ankle–foot dysfunction may have also contributed to postural instability (Table 2). Evidence supports that the loss of muscle quality and strength related to diabetes can also affect the foot–ankle function [36], with reduced strength of ankle flexors and ankle extensors [37], lower peak dorsiflexion range of motion and higher passive ankle stiffness [38]. Additionally, muscle dysfunction could be multifactorial, including the excess weight with derangement of carbohydrate and lipid metabolism [39].

In the year 2015, a systematic evaluation of the health effects of high B.M.I. in 195 countries showed that diabetes was the main cause of years lived with disability (17.1 million; 95% C.I. 10.6–24.4), followed by musculoskeletal disorders (5.7 million; 95% C.I. 3.4–8.8) [40]. Even in the absence of diabetes, increased B.M.I. is associated with fat infiltration in leg skeletal muscles [41,42] and peripheral nerve impairment [43], with a complex interaction among obesity, insulin resistance, chronic inflammation, and muscle loss [44].

Sarcopenia affects approximately 40% to 45% adults with overweight and obesity, increasing the risk of diabetes by circa 38% [45]. Among patients with diabetes, sarcopenic obesity, which indicates reduced lean body mass in the context of excess adiposity [46], has an estimated global prevalence of 27%, with higher prevalence in females compared to males [47].

Nonetheless, several studies have shown that obesity can promote biological aging (for review [48]); while sarcopenic obesity is related to a double aged phenotype, with an aging phenotype hidden within obese physiology (for review [49]). In early adulthood, obesity is associated with the expression of biochemical aging markers [50]. Likewise, diabetes is a cause of accelerate aging [51,52], with increased telomere shortening and mitochondrial DNA depletion [52]. Hence, diabetes and obesity can accelerate the aging process in multiple organs with functional loss and reduction in muscle function (for review, [53]). Consistently, in this study, the influence of age on postural sway faded in participants with diabetes who also had decreased muscle strength.

In each group of participants of this study, but mainly in participants with diabetes, the contribution of a history of COVID-19 to postural instability was also evident. This result agrees with the muscle weakness reported by patients during both the acute phase [54] and chronic phase [13] of COVID-19. Consistently, patients with diabetes who had SARS-CoV-2 infection, compared to those without infection, may have more fatigue after the acute phase of the disease, which is related to reduced handgrip strength [55]. Although etiology remains obscure, current knowledge suggests that metabolic and inflammatory factors may be involved (for review, [56]). In addition, a potential cofactor that cannot be discarded is the exposure to the COVID-19 mRNA vaccine. In this study, obesity prevailed among participants with diabetes and it has been associated with reduced adaptive response to the COVID-19 mRNA vaccine, while metabolic improvement can reverse this effect [57].

The results of this study need to be interpreted with caution due to the limitations of the design. The cross-sectional design precludes causal inferences, while the sample size allowed the observation of mild to moderate correlations amid the study variables without denying other relationships. Since patients were recruited within a healthcare institution, using very stringent criteria, the outcomes could vary in different contexts. The selection of participants with diabetes among those attending primary care, as well as no severe/critical COVID-19 cases, restrain the clinical transference of the results. The selection of participants without diabetes among relatives or companions of the patients counteracted the potential influence of sociodemographic and cultural cofactors (such as access to healthcare and nutrition); however, it also limited the generalizability of the results. The low frequency of the use of statins in the sample impeded the analysis of their potential effect on muscle strength, while no standardized record on the type of COVID-19 vaccination impeded further consideration of this variable.

The strengths of the study include the wide age range of the participants, with a consecutive sampling, as well as the prospective design with the collection of data by a team of trained health professionals. However, larger studies in less homogenous populations are required to adequately assess varied cofactors and translate the results into clinical practice.

## 5. Conclusions

Diabetes can modify the influence of individual cofactors contributing to postural sway, with a distorted influence of age, and reduced ankle movement. In adults with diabetes, a history of mild to moderate COVID-19 may have influence on the postural control in altered sensory conditions. The results are worth considering when assessing postural control in adults with diabetes. Further research needs to be conducted in clinical settings to translate these findings into patient benefit.

## Figures and Tables

**Table 1:** General characteristics of the participants by group and gender. Comparisons were performed between all participants in the groups, according to data distribution (using “*t*” test or x^2^). NA= Not Applicable.

		Diabetes (*n* = 99)			No Diabetes (*n* = 164)		
Variable	Men	Women	All	Men	Women	All	(t or x^2^) *p*
Number of participants	38	61	99	69	95	164	-
Years of age (mean ± S.D.)	58.2 ± 11.5	58.8 ± 8.9	58.6 ± 9.9	56.5 ± 10.4	60.0 ± 10.1	58.5 ± 10.3	(0.58) 0.55
Time since diagnosis (mean ± S.D.)	11.2 ± 9.6	7.6 ± 7.1	9.0 ± 8.3	NA	NA	NA	-
Body mass index (mean ± S.D.)	29.7 ± 5.5	29.7 ± 4.6	29.7 ± 4.9	29.3 ± 4.6	27.6 ± 4.0	28.3 ± 4.3	(2.28) 0.023
Right quadriceps strength (kg)	37.7 ± 9.4	19.6 ± 7.2	26.5 ± 12.0	42.8 ± 10.1	26.0 ± 8.0	31.8 ± 12.1	(3.40) 0.0007
Left quadriceps strength (kg)	37.7 ± 10.3	19.5 ± 6.8	26.5 ± 12.1	42.1 ± 10.1	24.8 ± 7.6	30.7 ± 12.5	(2.70) 0.007
Spectacles due to refraction errors (*n*, %)	15 (40%)	14 (23%)	29 (29%)	30 (44%)	39 (42%)	69 (42%)	(4.45) 0.34
Tobacco smokers (*n*, %)	6 (15%)	6 (9%)	12 (12%)	10 (14%)	11 (11%)	21 (12%)	(0.0) 1.00
Alcohol use (*n*, %)	11 (28%)	4 (6%)	15 (15%)	21 (30%)	19 (20%)	40 (24%)	(3.04) 0.081
History of COVID-19 (*n*, %)	24 (63%)	29 (47%)	53 (53%)	33 (47%)	48 (50%)	81 (49%)	(0.39) 0.53
Systemic high blood pressure (*n*, %)	16 (42%)	17 (27%)	33 (33%)	10 (14%)	23 (24%)	33 (20%)	(5.55) 0.018
Dyslipidemia (*n*, %)	4 (10%)	12 (19%)	16 (16%)	6 (8%)	8 (8%)	14 (8%)	(4.01) 0.045
Peripheral neuropathy (*n*, %)	20 (54%)	19 (33%)	39 (39%)	0 (0%)	0 (0%)	0 (0%)	(74.68) <0.0001

**Table 2:** The median and quartiles 1 and 3 of posturography measurements during the 8 sensory conditions, by group and gender. Comparisons were made between all participants of each group. The conditions were: 1. hard surface/eyes open, 2. hard surface/eyes closed, 3. hard surface/eyes open, 4. hard surface/eyes open/30° neck extension, 5. soft surface/eyes open, 6. soft surface/eyes closed, 7. soft surface/eyes open, 8. soft surface/eyes open/30° neck extension.

		Diabetes			No Diabetes		
Variables	Men (*n* = 38)	Women (*n* = 61)	All (*n* = 99)	Men (*n* = 69)	Women (*n* = 95)	All (*n* = 164)	*p* (Z Value)
1 Area	175 (122–231)	126 (79–186)	142 (17–620)	118 (79–186)	95 (62–135)	100 (13–565)	0.0003 (−3.59)
2 Area	249.5 (135–354)	138 (81–225)	167 (15–2382)	176 (116–273)	100 (65–162)	132 (17–974)	0.003 (−2.88)
3 Area	117.5 (74–169)	79 (54–140)	96 (15–840)	101 (60–144)	63 (38–98)	76 (10–342)	0.004 (−2.82)
4 Area	165.5 (94–283)	92 (65–162)	121 (15–1167)	98 (63–143)	72 (38–116)	81 (13–861)	0.0001 (−3.74)
5 Area	233 (171–404)	167 (113–278)	203 (46–1572)	194 (135–293)	131 (85–180)	150 (38–637)	0.001 (−3.19)
6 Area	534 (327–752)	344 (217–498)	401 (42–2996)	362 (233–523)	219 (144–370)	262 (43–1398)	<0.00001 (−4.07)
7 Area	259.5 (152–456)	148 (102–316)	202 (36–1579)	181 (96–266)	139 (80–205)	146 (36–768)	0.0002 (−4.12)
8 Area	278.5 (171–432)	188 (114–329)	213 (38–1310)	190 (107–288)	134 (87–180)	150 (38–761)	<0.00001 (−2.52)
1 Length	437 (392–533)	360 (315–431)	389 (170–1042)	378 (300–467)	329 (271–422)	347 (60–777)	0.011 (−1.89)
2 Length	667 (504–765)	458 (365–636)	558 (202–2305)	566 (459–708)	435 (325–5599)	479 (203–3184)	0.058 (−2.60)
3 Length	413 (323–522)	324 (273–398)	336 (143–1319)	336 (290–426)	302 (240–359)	311 (180–857)	0.0009 (−2.60)
4 Length	435 (321–593)	353 (290–475)	385 (153–1678)	353 (295–451)	308 (257–403)	323 (191–1062)	0.001 (−3.11)
5 Length	561 (445–753)	428 (382–559)	476 (229–1768)	479 (380–569)	397 (335–501)	421 (223–921)	0.004 (−2.82)
6 Length	897 (685–1277)	624 (496–791)	726 (233–2632)	734 (592–995)	602 (493–737)	642 (247–1952)	0.098 (−1.65)
7 Length	523 (398–698)	397 (350–496)	446 (195–1482)	436 (335–529)	360 (300–422)	375 (205–1023)	0.001 (−3.27)
8 Length	574 (449–765)	428 (360–560)	484 (205–1546)	449 (354–574)	380 (328–459)	409 (210–1097)	0.0007 (−3.38)
1 L-M position	−3.5 (−6.3–1.6)	0.8 (−3–3.3)	−0.8 (−13.1–11.2)	−2.2 (−5.5–2.3)	−0.8 (−3.6–3.3)	−1.0 (−16.3–11.9)	0.82 (−0.21)
2 L-M position	−3.3 (−7.2–0.9)	0.6 (−3.9–3.9)	−1.3 (−17.1–16.6)	−2.4 (−5.8–2.3)	−0.3 (−3.6–2.7)	−1.28 (−16.3–12.8)	0.93 (−0.08)
3 L-M position	−2.6 (−8.7–2.4)	0.1 (−4.7–4.2)	−0.5 (−14.8–17.2)	−1.8 (−6.3–4.6)	−0.3 (−4.1–4.2)	−1.2 (−13.6–16.4)	0.78 (0.27)
4 L-M position	−2.6 (−6.1–3)	−0.1 (−5.4–3)	−0.9 (−18.7–15.7)	−2.2 (−5.6–2.4)	−1.4 (−5–3.4)	−1.7 (−19.9–18.5)	0.81 (0.23)
5 L-M position	−0.65 (−5.3–5)	0.6 (−3.8–6.8)	0 (−21.6–17.1)	−1.8 (−4.9–1.6)	−0.6 (−4.6–3.9)	−1.2 (−14.6–15.1)	0.14 (−1.46)
6 L-M position	−0.7 (−6.9–7)	0.9 (−5.3–7.1)	0.5 (−19–16.2)	−2.1 (−6.2–2.8)	0.1 (−3.8–4.2)	−0.6 (−16.5–18.4)	0.28 (−1.07)
7 L-M position	−1.7 (−8.8–4.8)	0.7 (−4.4–5.6)	0 (−18.5–33.1)	−2.2 (−6.4–3.4)	−1.3 (−5.1–2.7)	−1.6 (−18.4–20)	0.22 (−1.20)
8 L-M position	−0.4 (−7.7–6.9)	0.1 (−3.1–5.8)	0.1 (−17.3–18.5)	−2 (−6.5–3.7)	−1.4 (−4.8–3.7)	−1.5 (−14.8–26.3)	0.14 (−1.45)
1 A-P position	−49.7 (−64.2–−40.6)	−36.5 (−45.3–−26.6)	−41.6 (−87.1–−8.9)	−51.8 (−60.7–−41.7)	−34.7 (−42–−25.1)	−40.8 (−91.4–5.4)	0.52 (0.64)
2 A-P position	−43 (−59.8–−35.6)	−35.5 (−44–−25.3)	−38 (−73.7–−3)	−46.6 (−56.3–−36.4)	−29.9 (−36.9–−21.7)	−35 (−78.1–−2.8)	0.15 (1.41)
3 A-P position	−47.85 (−61.1–−35.2)	−37.1 (−45.7–−29.3)	−39.6 (−81.3–−2.5)	−45.7 (−58.1–−37.9)	−31.5 (−38.7–−22.7)	−37.9 (−83.6–−2.3)	0.11 (1.55)
4 A-P position	−48.8 (−53.9–−42.2)	−36.9 (−46–−28)	−41.7 (−79.3–1.3)	−47.1 (−57.8–−38.9)	−33.5 (−40.3–−23.4)	−37.5 (−89–−7.6)	0.17 (1.36)
5 A-P position	−53 (−62.6–−34.7)	−34.4 (−40.6–−27.1)	−38.6 (−83.4–8)	−49.8 (−62.7–−37.5)	−29.6 (−38.6–−19.9)	−37.4 (−95.8–8.1)	0.11 (1.58)
6 A-P position	−50.2 (−61.4–−42.1)	−31.6 (−41–−21.4)	−35 (−78.7–9.1)	−46 (−58–−35.5)	−26.3 (−34.7–−18.3)	−33.8 (−90.2–7.9)	0.25 (1.12)
7 A-P position	−52.6 (−65.9–−39.7)	−35.8 (−42.9–−27.3)	−39.7 (−85.3–7.7)	−48.3 (−62–−39.3)	−30.3 (−38.6–−20.3)	−37.5 (−99.8–2.1)	0.18 (1.32)
8 A-P position	−51.4 (−66.4–−36.7)	−36.3 (−44–−28.6)	−38.4 (−85.3–6.8)	−49.4 (−64.1–−39.7)	−32.7 (−41.1–−21.7)	−39.1 (−100–0)	0.48 (0.69)
1 VFY	−7.9 (−13.2–−5.9)	−6.9 (−9.5–−4.1)	−7.6 (−20.4–1.29)	−10.2 (−13.5–−8.0)	−6.34 (−8.7–−3.6)	−8.1 (−22.4–4.0)	0.55 (−0.59)
2 VFY	−1.9 (−9.9–2.8)	−3.5 (−8.1–0.4)	−2.6 (−15.7–48.4)	−3.9 (−8.6–−0.4)	−3.36 (−5.4–1.4)	−3.5 (−19.9–53.7)	0.83 (0.20)
3 VFY	−8.6 (−13.5–−5.5)	−7.6 (−9.7–−4.6)	−7.8 (−18.9–9.9)	−9.8 (−12.9–−7.3)	−6.92 (−8.8–−4.2)	−7.9 (−21.2–12.0)	0.90 (−0.11)
4 VFY	−7.2 (−11.6–−0.9)	−6.1 (−9.4–−2.1)	−6.6 (−23.6–18.7)	−8.6 (−12.9–−4.1)	−5.2 (−8.6–−0.3)	−6.7 (−23.5–11.8)	0.77 (−0.28)
5 VFY	−9.7 (−12.2–−6.9)	−7.8 (−9.3–−6.4)	−8.1 (−16.4–11.2)	−11.3 (−12.9–−9.4)	−8.27 (−9.6–−6.9)	−9.2 (−19.3–7)	0.010 (−2.54)
6 VFY	−1.5 (−9.3–6.8)	0.7 (−4.4–5.9)	0.58 (−16.5–45.1)	−2.3 (−6.8–2.5)	0.7 (−3.4–5.7)	−0.3 (−20.5–31.2)	0.54 (−0.60)
7 VFY	−10.3 (−14.0–−8.2)	−8.5 (−9.8–−6.0)	−9.0 (−18.7–−13.6)	−11.8 (−13.7–−9.9)	−8.9 (−9.9–−7.4)	−9.7 (−19.9–0.1)	0.035 (−2.10)
8 VFY	−6.3 (−13.5–1.2)	−4.3 (−8.7–0.3)	−4.7 (−21.4–−29.0)	−7.7 (−12.6–−2.9)	−3.7 (−8.3–0.4)	−5.7 (−24.0–16.9)	0.43 (−0.78)

L-M = latero-medial position of the center of pressure; A-P = anterior–posterior position of the center of pressure; VFY = velocity as a function of the anterior–posterior position of the center of pressure.

**Table 3:** Repeated measures multivariate analysis of covariance on the posturography measurements, in all sensory conditions of 99 adults with diabetes. L.L. = lateral–lateral. A.P. = anterior–posterior. V.F.Y. = velocity as a function of the anterior–posterior position of the center of pressure. B.M.I. = Body Mass Index. d.f. = degrees of freedom. N.S. = not significant.

Contributing Variables	Sway Area	Sway Length	L.L. Position	A.P. Position	V.F.Y.
Multiple R (*p* Value)	R = 0.37–0.42 (≤0.02) ^1^	R = 0.44–0.55 (<0.0005)	N.S.	R = 0.39–0.57 (<0.01)	R= 0.34–0.43 (<0.02) ^2^
	*p* (F) (d.f. 1, 7, 92)	*p* (F) (d.f. 1, 7, 92)	*p* (F) (d.f. 1, 7, 92)	*p* (F) (d.f. 1, 7, 92)	*p* (F) (d.f. 1, 7, 92)
Intercept	0.17 (1.85)	0.039 (4.35)	0.60 (0.27)	0.19 (1.72)	0.91 (0.01)
Age	0.61 (0.25)	0.08 (3.11)	0.49 (0.46)	0.59 (0.28)	0.90 (0.01)
B.M.I.	0.32 (0.96)	0.27 (1.20)	0.91 (0.01)	0.038 (4.40)	0.72 (0.12)
Quadriceps strength	0.011 (6.67)	0.0004 (13.24)	0.90 (0.01)	0.61 (0.25)	0.048 (3.99)
Gender	0.003 (8.72)	<0.0001(24.09)	0.35 (0.87)	0.0003 (13.90)	0.77 (0.08)
History of COVID-19	0.029 (4.90)	0.001 (10.40)	0.018 (5.78)	0.64 (0.20)	0.07 (3.32)
Gender * COVID-19	0.57 (0.32)	0.39 (0.71)	0.72 (0.12)	0.54 (0.37)	0.76 (0.08)
Repeated measures (R1)	0.42 (1.00)	0.69 (0.66)	0.79 (0.54)	0.89 (0.41)	0.42 (1.00)
R1 * Age	0.92 (0.35)	0.69 (0.66)	0.41 (1.01)	0.64 (0.73)	0.57 (0.81)
R1 * B.M.I.	0.38 (1.07)	0.38 (1.06)	0.93 (0.33)	0.91 (0.37)	0.65 (0.71)
R1 * Strength	0.08 (1.81)	0.002 (3.22)	0.97 (0.24)	0.81 (0.53)	0.41 (1.02)
R1 * Gender	0.19 (1.41)	<0.0001 (6.53)	0.99 (0.12)	0.093 (1.75)	0.35 (1.10)
R1 * COVID-19	0.19 (1.41)	0.020 (2.38)	0.14 (1.57)	0.93 (0.34)	0.70 (0.65)
R1 * Gender * COVID-19	0.99 (0.10)	0.95 (0.28)	0.47 (0.94)	0.68 (0.69)	0.70 (0.66)

^1^ Just for conditions 1. hard surface/eyes open, 3. hard surface/eyes open, 5. soft surface/eyes open, and 7. soft surface/eyes open. ^2^ Just for conditions 2. hard surface/eyes closed, 4. hard surface/eyes open/30° neck extension, and 5. soft surface/eyes open.

**Table 4:** Repeated measures multivariate analysis of covariance on the posturography measurements, in all sensory conditions, of 164 adults without diabetes.

Contributing Variables	Sway Area	Sway Length	L.L. Position	A.P. Position	V.F.Y.
Multiple R (*p* Value)	R = 0.27–0.35 (≤0.001)	R = 0.29–0.45 (≤0.03)	N.S.	R = 0.55–0.63 (<0.00001)	R = 0.40–0.59 (<0.00001)
	*p* (F) (d.f. 1, 7, 157)	*p* (F) (d.f. 1, 7, 157)	*p* (F) (d.f. 1, 7, 157)	*p* (F) (d.f. 1, 7, 157)	*p* (F) (d.f. 1, 7, 157)
Intercept	0.10 (2.64)	0.031 (4.70)	0.0348 (4.52)	0.007 (7.29)	0.048 (3.96)
Age	0.89 (0.01)	<0.0001 (16.78)	0.28 (1.15)	0.033 (4.61)	0.0002 (14.23)
B.M.I.	0.38 (0.77)	0.14 (2.12)	0.32 (0.96)	0.007 (7.43)	0.004 (8.45)
Quadriceps strength	0.72 (0.12)	0.32 (0.98)	0.059 (3.61)	0.35 (0.86)	0.60 (0.26)
Gender	0.008 (7.018)	0.041 (4.22)	0.024 (5.13)	<0.0001(28.38)	0.004 (8.31)
History of COVID-19	0.21 (1.57)	0.13 (2.23)	0.41 (0.67)	0.79 (0.067)	0.27 (1.21)
Gender * COVID-19	0.057 (3.65)	0.92 (0.009)	0.89 (0.01)	0.941 (0.005)	0.90 (0.01)
Repeated measures (R1)	0.001 (3.36)	0.022 (2.34)	0.62 (0.75)	0.29 (1.20)	0.003 (3.06)
R1 * Age	0.16 (1.49)	<0.0001 (4.42)	0.77 (0.57)	0.62 (0.75)	0.0001 (4.20)
R1 * B.M.I.	0.15 (1.52)	0.0003 (3.88)	0.78 (0.57)	0.28 (1.23)	0.001 (3.36)
R1 * Strength	0.95 (0.29)	0.21 (1.37)	0.41 (1.02)	0.96 (0.28)	0.129 (1.60)
R1 * Gender	0.023 (2.31)	0.003 (3.05)	0.25(1.28)	0.09 (1.72)	0.286 (1.22)
R1 * COVID-19	0.79 (0.55)	0.0001 (4.29)	0.004 (2.94)	0.007 (2.75)	0.262 (1.26)
R1 * Gender * COVID-19	0.40 (1.03)	0.95 (0.29)	0.98(0.21)	0.59 (0.79)	0.76 (0.58)

L.L. = lateral–lateral. A.P. = anterior–posterior. V.F.Y. = velocity as a function of the anterior–posterior position of the center of pressure. B.M.I. = Body Mass Index. d.f. = degrees of freedom. N.S. = not significant.

**Table 5:** Repeated measures multivariate analysis of covariance on the posturography measurements, in all sensory conditions of 263 matched adults (99 with/164 without diabetes).

Contributing Variables	Sway Area	Sway Length	L.L. Position	A.P. Position	V.F.Y.
Multiple R (*p* Value)	R = 0.28–0.34(≤0.001)	R = 0.34–0.41(<0.00001)	N.S.	R = 0.5–0.60(<0.00001)	R = 0.28–0.57 (<0.00001)
	*p* (F) (d.f. 1, 7, 256)	*p* (F) (d.f. 1, 7, 256)	*p* (F) (d.f. 1, 7, 256)	*p* (F) (d.f. 1, 7, 256)	*p* (F) (d.f. 1, 7, 256)
Intercept	0.035 (4.46)	0.010 (6.65)	0.036 (4.40)	0.001 (10.21)	0.06 (3.34)
Age	0.64 (0.21)	<0.0001 (17.75)	0.15 (2.00)	0.18 (1.76)	0.004 (8.04)
B.M.I.	0.268 (1.23)	0.94 (0.005)	0.42 (0.62)	0.001 (10.62)	0.042 (4.14)
Quadriceps strength	0.079 (3.09)	0.17 (1.83)	0.08 (3.05)	0.76 (0.08)	0.14 (2.18)
Gender	0.0002 (14.03)	<0.0001 (22.15)	0.016 (5.87)	<0.0001 (39.76)	0.06 (3.42)
Diabetes	<0.0001 (20.27)	0.0004 (12.44)	0.41 (0.66)	0.30 (1.05)	0.26 (1.26)
Gender * Diabetes	0.88 (0.01)	0.10 (2.71)	0.70 (0.14)	0.37 (0.79)	0.28 (1.17)
Repeated measures (R1)	0.002 (3.09)	0.034 (2.16)	0.49 (0.91)	0.64 (0.72)	0.001 (3.35)
R1 * Age	0.49 (0.91)	0.0001 (4.06)	0.46 (0.95)	0.40 (1.03)	0.0007 (3.61)
R1 * B.M.I.	0.61 (0.76)	0.21 (1.37)	0.79 (0.54)	0.93 (0.34)	0.30 (1.19)
R1 * Strength	0.67 (0.69)	0.91 (0.37)	0.59 (0.79)	0.97 (0.25)	0.09 (1.75)
R1 * Gender	0.027 (2.25)	<0.0001 (8.03)	0.72 (0.63)	0.0003 (3.83)	0.049 (2.02)
R1 * Diabetes	<0.0001 (6.69)	0.024 (2.30)	0.111 (1.67)	0.85 (0.47)	0.62 (0.75)
R1 * Gender * Diabetes	0.95 (0.28)	0.86 (0.46)	0.96 (0.27)	0.26 (1.25)	0.69 (0.67)

L.L. = lateral–lateral. A.P. = anterior–posterior. V.F.Y. = velocity as a function of the anterior–posterior position of the center of pressure. B.M.I. = Body Mass Index. d.f. = degrees of freedom. N.S. = not significant.

## Data Availability

All data generated or analyzed during this study are included in this article. Further enquiries can be directed to the corresponding author.

## Abbreviations

The following abbreviations are used in this manuscript:

L.L.	Lateral–lateral
A.P.	Anterior–posterior
B.M.I.	Body Mass Index
d.f.	Degrees of freedom
N.S.	Not significant
V.F.Y.	Velocity as a function of the anterior–posterior position of the center of pressure
95% C.I.	95% Confidence Interval
