# Correction: Jáuregui-Renaud et al. Early Postural Instability with History of COVID-19 Influence Related to Diabetes: An Exploratory Cross-Sectional Study. *J. Clin. Med.* 2026, *15*, 3178

**DOI:** 10.3390/jcm15208188

**Published:** 2026-10-21

**Authors:** Kathrine Jáuregui-Renaud, José Adán Miguel-Puga, Aida García-López, María de Lourdes Tirado-Mondragón

**Affiliations:** 1 Unidad de Investigación Médica en Otoneurología, Instituto Mexicano del Seguro Social, Ciudad de México 06720, Mexicodraidagarcia@gmail.com (A.G.-L.); 2 Hospital General de Zona 8, Instituto Mexicano del Seguro Social, Ciudad de México 01090, Mexico

## Error in Table

In the original publication [1], there was a mistake in the first column of Table 2 that had a missing value, and negative signs were missing. The correct table is below. The authors state that the scientific conclusions are unaffected. This correction was approved by the Academic Editor. The original publication has also been updated.

**Table 2:** The median and quartiles 1 and 3 of posturography measurements during the 8 sensory conditions, by group and gender. Comparisons were made between all participants of each group. The conditions were: 1. hard surface/eyes open, 2. hard surface/eyes closed, 3. hard surface/eyes open, 4. hard surface/eyes open/30° neck extension, 5. soft surface/eyes open, 6. soft surface/eyes closed, 7. soft surface/eyes open, 8. soft surface/eyes open/30° neck extension.

		Diabetes			No Diabetes		
Variables	Men (*n* = 38)	Women (*n* = 61)	All (*n* = 99)	Men (*n* = 69)	Women (*n* = 95)	All (*n* = 164)	*p* (Z Value)
1 Area	175 (122–231)	126 (79–186)	142 (17–620)	118 (79–186)	95 (62–135)	100 (13–565)	0.0003 (−3.59)
2 Area	249.5 (135–354)	138 (81–225)	167 (15–2382)	176 (116–273)	100 (65–162)	132 (17–974)	0.003 (−2.88)
3 Area	117.5 (74–169)	79 (54–140)	96 (15–840)	101 (60–144)	63 (38–98)	76 (10–342)	0.004 (−2.82)
4 Area	165.5 (94–283)	92 (65–162)	121 (15–1167)	98 (63–143)	72 (38–116)	81 (13–861)	0.0001 (−3.74)
5 Area	233 (171–404)	167 (113–278)	203 (46–1572)	194 (135–293)	131 (85–180)	150 (38–637)	0.001 (−3.19)
6 Area	534 (327–752)	344 (217–498)	401 (42–2996)	362 (233–523)	219 (144–370)	262 (43–1398)	<0.00001 (−4.07)
7 Area	259.5 (152–456)	148 (102–316)	202 (36–1579)	181 (96–266)	139 (80–205)	146 (36–768)	0.0002 (−4.12)
8 Area	278.5 (171–432)	188 (114–329)	213 (38–1310)	190 (107–288)	134 (87–180)	150 (38–761)	<0.00001 (−2.52)
1 Length	437 (392–533)	360 (315–431)	389 (170–1042)	378 (300–467)	329 (271–422)	347 (60–777)	0.011 (−1.89)
2 Length	667 (504–765)	458 (365–636)	558 (202–2305)	566 (459–708)	435 (325–5599)	479 (203–3184)	0.058 (−2.60)
3 Length	413 (323–522)	324 (273–398)	336 (143–1319)	336 (290–426)	302 (240–359)	311 (180–857)	0.0009 (−2.60)
4 Length	435 (321–593)	353 (290–475)	385 (153–1678)	353 (295–451)	308 (257–403)	323 (191–1062)	0.001 (−3.11)
5 Length	561 (445–753)	428 (382–559)	476 (229–1768)	479 (380–569)	397 (335–501)	421 (223–921)	0.004 (−2.82)
6 Length	897 (685–1277)	624 (496–791)	726 (233–2632)	734 (592–995)	602 (493–737)	642 (247–1952)	0.098 (−1.65)
7 Length	523 (398–698)	397 (350–496)	446 (195–1482)	436 (335–529)	360 (300–422)	375 (205–1023)	0.001 (−3.27)
8 Length	574 (449–765)	428 (360–560)	484 (205–1546)	449 (354–574)	380 (328–459)	409 (210–1097)	0.0007 (−3.38)
1 L-M position	−3.5 (−6.3–1.6)	0.8 (−3–3.3)	−0.8 (−13.1–11.2)	−2.2 (−5.5–2.3)	−0.8 (−3.6–3.3)	−1.0 (−16.3–11.9)	0.82 (−0.21)
2 L-M position	−3.3 (−7.2–0.9)	0.6 (−3.9–3.9)	−1.3 (−17.1–16.6)	−2.4 (−5.8–2.3)	−0.3 (−3.6–2.7)	−1.28 (−16.3–12.8)	0.93 (−0.08)
3 L-M position	−2.6 (−8.7–2.4)	0.1 (−4.7–4.2)	−0.5 (−14.8–17.2)	−1.8 (−6.3–4.6)	−0.3 (−4.1–4.2)	−1.2 (−13.6–16.4)	0.78 (0.27)
4 L-M position	−2.6 (−6.1–3)	−0.1 (−5.4–3)	−0.9 (−18.7–15.7)	−2.2 (−5.6–2.4)	−1.4 (−5–3.4)	−1.7 (−19.9–18.5)	0.81 (0.23)
5 L-M position	−0.65 (−5.3–5)	0.6 (−3.8–6.8)	0 (−21.6–17.1)	−1.8 (−4.9–1.6)	−0.6 (−4.6–3.9)	−1.2 (−14.6–15.1)	0.14 (−1.46)
6 L-M position	−0.7 (−6.9–7)	0.9 (−5.3–7.1)	0.5 (−19–16.2)	−2.1 (−6.2–2.8)	0.1 (−3.8–4.2)	−0.6 (−16.5–18.4)	0.28 (−1.07)
7 L-M position	−1.7 (−8.8–4.8)	0.7 (−4.4–5.6)	0 (−18.5–33.1)	−2.2 (−6.4–3.4)	−1.3 (−5.1–2.7)	−1.6 (−18.4–20)	0.22 (−1.20)
8 L-M position	−0.4 (−7.7–6.9)	0.1 (−3.1–5.8)	0.1 (−17.3–18.5)	−2 (−6.5–3.7)	−1.4 (−4.8–3.7)	−1.5 (−14.8–26.3)	0.14 (−1.45)
1 A-P position	−49.7 (−64.2–−40.6)	−36.5 (−45.3–−26.6)	−41.6 (−87.1–−8.9)	−51.8 (−60.7–−41.7)	−34.7 (−42–−25.1)	−40.8 (−91.4–5.4)	0.52 (0.64)
2 A-P position	−43 (−59.8–−35.6)	−35.5 (−44–−25.3)	−38 (−73.7–−3)	−46.6 (−56.3–−36.4)	−29.9 (−36.9–−21.7)	−35 (−78.1–−2.8)	0.15 (1.41)
3 A-P position	−47.85 (−61.1–−35.2)	−37.1 (−45.7–−29.3)	−39.6 (−81.3–−2.5)	−45.7 (−58.1–−37.9)	−31.5 (−38.7–−22.7)	−37.9 (−83.6–−2.3)	0.11 (1.55)
4 A-P position	−48.8 (−53.9–−42.2)	−36.9 (−46–−28)	−41.7 (−79.3–1.3)	−47.1 (−57.8–−38.9)	−33.5 (−40.3–−23.4)	−37.5 (−89–−7.6)	0.17 (1.36)
5 A-P position	−53 (−62.6–−34.7)	−34.4 (−40.6–−27.1)	−38.6 (−83.4–8)	−49.8 (−62.7–−37.5)	−29.6 (−38.6–−19.9)	−37.4 (−95.8–8.1)	0.11 (1.58)
6 A-P position	−50.2 (−61.4–−42.1)	−31.6 (−41–−21.4)	−35 (−78.7–9.1)	−46 (−58–−35.5)	−26.3 (−34.7–−18.3)	−33.8 (−90.2–7.9)	0.25 (1.12)
7 A-P position	−52.6 (−65.9–−39.7)	−35.8 (−42.9–−27.3)	−39.7 (−85.3–7.7)	−48.3 (−62–−39.3)	−30.3 (−38.6–−20.3)	−37.5 (−99.8–2.1)	0.18 (1.32)
8 A-P position	−51.4 (−66.4–−36.7)	−36.3 (−44–−28.6)	−38.4 (−85.3–6.8)	−49.4 (−64.1–−39.7)	−32.7 (−41.1–−21.7)	−39.1 (−100–0)	0.48 (0.69)
1 VFY	−7.9 (−13.2–−5.9)	−6.9 (−9.5–−4.1)	−7.6 (−20.4–1.29)	−10.2 (−13.5–−8.0)	−6.34 (−8.7–−3.6)	−8.1 (−22.4–4.0)	0.55 (−0.59)
2 VFY	−1.9 (−9.9–2.8)	−3.5 (−8.1–0.4)	−2.6 (−15.7–48.4)	−3.9 (−8.6–−0.4)	−3.36 (−5.4–1.4)	−3.5 (−19.9–53.7)	0.83 (0.20)
3 VFY	−8.6 (−13.5–−5.5)	−7.6 (−9.7–−4.6)	−7.8 (−18.9–9.9)	−9.8 (−12.9–−7.3)	−6.92 (−8.8–−4.2)	−7.9 (−21.2–12.0)	0.90 (−0.11)
4 VFY	−7.2 (−11.6–−0.9)	−6.1 (−9.4–−2.1)	−6.6 (−23.6–18.7)	−8.6 (−12.9–−4.1)	−5.2 (−8.6–−0.3)	−6.7 (−23.5–11.8)	0.77 (−0.28)
5 VFY	−9.7 (−12.2–−6.9)	−7.8 (−9.3–−6.4)	−8.1 (−16.4–11.2)	−11.3 (−12.9–−9.4)	−8.27 (−9.6–−6.9)	−9.2 (−19.3–7)	0.010 (−2.54)
6 VFY	−1.5 (−9.3–6.8)	0.7 (−4.4–5.9)	0.58 (−16.5–45.1)	−2.3 (−6.8–2.5)	0.7 (−3.4–5.7)	−0.3 (−20.5–31.2)	0.54 (−0.60)
7 VFY	−10.3 (−14.0–−8.2)	−8.5 (−9.8–−6.0)	−9.0 (−18.7–−13.6)	−11.8 (−13.7–−9.9)	−8.9 (−9.9–−7.4)	−9.7 (−19.9–0.1)	0.035 (−2.10)
8 VFY	−6.3 (−13.5–1.2)	−4.3 (−8.7–0.3)	−4.7 (−21.4–−29.0)	−7.7 (−12.6–−2.9)	−3.7 (−8.3–0.4)	−5.7 (−24.0–16.9)	0.43 (−0.78)

L-M = latero-medial position of the center of pressure; A-P = anterior–posterior position of the center of pressure; VFY = velocity as a function of the anterior–posterior position of the center of pressure.
